# Supplementary material for: The Virome of Babaco (Vasconcellea × heilbornii) Expands to Include New Members of the Rhabdoviridae and Bromoviridae
Source: Viruses. 2023 Jun 16;15(6):1380. doi: 10.3390/v15061380 (PMC10304175; doi:10.3390/v15061380)
Supplement: Supplementary file 1 [file viruses-15-01380-s001.zip › Supplementary Table S1.pdf]

Supplementary Table S1. Primer information for detection of babaco viruses.

	Primer information (5' – 3')				
**Virus**	**Forward**	**Reverse**	**Annealing temperature °C**	**Amplification product**	**Reference**
Papaya ringspot virus	GAGARGTAYATGCCGCGGTATGG	CGCATACCCAGGAGAGAGTGC	55	263	Quito-Avila et al. 2015
Babaco mosaic virus	GGATGCACTCATTACATCCAAGC	CCACTCCAAGGCTTCCATGAGC	57	647	Alvarez-Quinto et al. 2017
Babaco virus Q	CGTGTGCTTGCTGGTTTTCGTTC	CAACGGGAAACCCATACACCTGG	55 - 57	1055	Cornejo-Franco et al. 2020
Babaco cheravirus-1	GCTTGTCATTAGCACGGCTAAC	GCAGGAAAGAGCGTCTGATCA	55 - 57	447	Cornejo-Franco et al. 2020
Babaco nepovirus -1	GGTATGCTCGACAGAGCATTGT	CCCTTCTACATTCCACAACCAC	55 - 57	269	Cornejo-Franco et al. 2020
Babaco cryptic virus-1	GGACTAGTACACCCTACCAACG	CCATAGGGTACCATGCACAAAC	55 - 57	469	Cornejo-Franco et al. 2020
Babaco endogenous pararetrovirus	CACCCTTGGCGAATATATGCAG	TGCTCAACATGTCCTGAAGC	55 - 57	407	Cornejo-Franco et al. 2020
