# Supplementary material for: The Virome of Babaco (Vasconcellea × heilbornii) Expands to Include New Members of the Rhabdoviridae and Bromoviridae
Source: Viruses. 2023 Jun 16;15(6):1380. doi: 10.3390/v15061380 (PMC10304175; doi:10.3390/v15061380)
Supplement: Supplementary file 1 [file viruses-15-01380-s001.zip › Supplementary Table S2.pdf]

Supplementary Table S2. List of primers used in this study for genome amplification and detection of babaco ilarvirus 1 (BabIV-1) and babaco rhabdovirus 1 (BabRV-1).

Virus	Primer name	Sequence 5′ to 3′	Target genome* position (amplicon size nt)
BabIV-1	1Race-5'end-1	CGTTGCTAAAGCATCATGATCC	1-56
	1Race-5'end-2	CGAGTGACTTGAACTGTCACTC	1-281
	RNA1-F1	CCAATCTCTCTCGTAATGGATC	18-866 (849)
	RNA1-R1	CCTCTCAATTCGGTAAGCCTT	
	RNA1-F2	GGTATGAGCACAAGTTTTCTGT	778-1697 (920)
	RNA1-R2	GGTTAGTTTCTCGTCTTTCAGC	
	RNA1-F3	GTGGTTCTGGAAACTACCTGG	1554-2968 (1415)
	RNA1-R3	GGGTCCTTACCACTGTATATGT	
	1Race-3'end-1	CGAATGTTGCACTGGTCCGTA	2908-3299 (392)
	1Race-3'end-2	CGTACCTGCACACTGATCATAAG	3115-3299 (185)
	2Race-5'end-1	CGAACAAGACCTTAGTGGCCG	1-59
	2Race-5'end-2	GGTCTAAGGAAAACTACTAACCT	1-186
	RNA2-F1	CCATCACGATTTGAGAGAATTCC	64-939 (876)
	RNA2-R1	CGTTTCGACTAACTCCTGGAA	
	RNA2-F2	CCAACAACATCCGACATACTCA	808-1612 (805)
	RNA2-R2	GATGTAACCTCTGTTGAGACTTG	
	RNA2-F3	GAGAGAGCATTACCAGCTACTA	1378-2605 (1228)
	RNA2-R3	CTCTATATCCACACGGTGGAG	
	2Race-3'end-1	CGAGTGATTCGTCGTATGGAGTGG	2342-2675 (334)
	2Race-3'end-2	CGAAAAGCAGAATGTGTTGAAC	2400-2675 (276)
	3Race-5'end-1	GATCTGGACCAGGAGAAGTAGT	1-159
	3Race-5'end-2	CGACCTACATCTCTATAGATCT	1-358
	RNA3-F1	GGAAGAGCTTCAGCAAGTCAC	152-1118 (967)
	RNA3-R1	CTTGAGCTCCAGTCGGATGAC	
	RNA3-F2	GGTTAGAAGCAGGATTGCGTC	722-1688 (967)
	RNA3-R2	CCTAGACCTCCAACAAGTCTTC	
	3Race-3'end-1	CGATTTGCACGAGAGTGCAGA	1526-1802 (277)
	3Race-3'end-2	CGTAGTAACGACTGAGTTACCACG	1609-1802 (194)
	Detection_F	GGTTAGAAGCAGGATTGCGTC	722-1118 (397)
	Detection_R	CTTGAGCTCCAGTCGGATGAC	

BabRV-1	Race-5’	CCCATCAGCTGAAGGAATTGC	1-251
	F1	GACTCTATGCCTACTGTGGTAC	80-1152 (1073)
	R1	CGACACTGAGATCAACAAAGTAG	
	F2	GCAACTGTTCATGACAATCCCA	1056-2062 (1007)
	R2	GGTGTCCTATCTCTCAGTTTCC	
	F3	CTATCAGTGCCTGAGCTGAACT	1962-2991 (1030)
	R3	GCAATTGCAATAGGAAGGACT	
	F4	CCTCAAAGATGTCTAAACCTCT	2907-3850 (944)
	R4	GTAGGTAGGATTAAGGCTGTAG	
	F5	GAGCATCGAGGAACACCCTCA	3793-4670 (878)
	R5	GATCGGATCCTTCTGATTTTCTC	
	F6	CCAGACTGAACATCATGGTCT	4563-5511 (949)
	R6	GACCAAACATCTGTGTCCATGG	
	F7	GACATGCTTGACCTATGTCTCA	5305-6233 (929)
	R7	CTGAGTGATCACAACTCATCTG	
	F8	GCAGATTGGGTTCATCAGATGT	6153-7059 (907)
	R8	CCATATCTCCAACAGTCTGTCT	
	F9	CCACGATGGAAGACTGGGAAG	6862-7930 (1069)
	R9	CCATCTATATCTATGATCGGATG	
	F10	GAGATGATATATCAGAGACAGGC	7795-8790 (996)
	R10	GGAGGTCGTGAGTGATGTCAAA	
	F11	GAAGTGGAATCAGCAAATGAGG	8687-9719 (1033)
	R11	GAATGACATTCCCATCAGTTGTC	
	F12	GAGAGCTACATATGATCAATCCC	9655-10570 (916)
	R12	CGATGATCATAGCTACCTCTGA	
	F13	CACAAACATTGATCCCTCACAAC	10505-11437 (933)
	R13	CCAAGACAATCTAAACAAGAGAG	
	F14	GCAGATTGTCCTTCATGTTATAG	11375-12359 (985)
	R14	CTCAATCTTCTCCAGTATTCTCT	
	F15	CCAGATACAAGTTCTTGAATAGG	12292-12765 (474)
	R15	GGTGGTGATAGGGATTAAGTCC	
	Race 3’	CCTAGACTGGTTCGCTTCTGAT	12712-12802 (91)
	Detection_F	GGAGTCGGCATTGGTATAGCA	1347-2269 (923)
	Detection_R	CCGTTGTCCTTTAACACCCTAG	

* For babaco rhabdovirus-1 the primer positions correspond to the complementary (positive-sense) genome.
