# Supplementary material for: The Virome of Babaco (Vasconcellea × heilbornii) Expands to Include New Members of the Rhabdoviridae and Bromoviridae
Source: Viruses. 2023 Jun 16;15(6):1380. doi: 10.3390/v15061380 (PMC10304175; doi:10.3390/v15061380)
Supplement: Supplementary file 1 [file viruses-15-01380-s001.zip › Supplementary Table S3.pdf]

Supplementary Table S3. Nucleotide (nt) and amino acid (aa) identities between open reading frames (ORFs) and their predicted products of babaco ilarvirus-1 (BabIV-1) and counterparts from all ilarviruses currently available at NCBI.

Ilarviruses	**RNA 1** nt	**ORF 1a** nt(aa)	**RNA 2** nt	**ORF2a** nt(aa)	**RNA 3** nt	**ORF3a** nt(aa)	**ORF3b** nt(aa)
**Subgroup 1**							
Ageratum latent virus	**39,88**	43,19 (51,26)	**36,29**	40,08 (43,56)	**21,78**	33,84 (33,11)	28,27 (26,77)
Parietaria mottle virus	**40,42**	42,34 (51,49)	**36,86**	39,56 (44,03)	**21,74**	31,32 (33,11)	28,80 (25,75)
Tobacco streak virus	**40,77**	42,83 (50,72)	**36,62**	39,00 (44,42)	**22,51**	31,86 (32,67)	29,21 (28,40)
Privet ringspot virus	**39,79**	42,24 (50,32)	**36,86**	39,72 (44,14)	**21,82**	32,45 (35,31)	28,32 (25,00)
Strawberry necrotic shock virus	**39,43**	42,31 (50,63)	**37,14**	39,38 (44,55)	**22,90**	31,97 (33,99)	27,99 (30,33)
Blackberry chlorotic ringspot virus	**39,69**	42,22 (50,32)	**36,25**	38,82 (44,20)	**23,70**	32,91 (33,99)	30,16 (28,19)
Average (% identity)	**40,00**	**42,52 (50,79)**	**36,67**	**39,43 (44,15)**	**22,41**	**32,39 (33,70)**	**28,79 (27,41)**
**Subgroup 2**							
Tulare apple mosaic virus	**39,85**	42,82 (48,80)	**33,48**	36,45 (40,35)	**21,90**	32,55 (34,90)	29,63 (27,04)
Citrus leaf rugose virus	**39,67**	42,97 (52,00)	**34,29**	37,02 (40,35)	**20,26**	32,67 (33,67)	31,28 (25,42)
Spinach latent virus	**39,49**	43,35 (49,22)	**34,45**	39,48 (40,53)	**23,79**	31,65 (34,34)	33,29 (29,44)
Elm mottle virus	**40,21**	43,46 (50,00)	**33,59**	38,19 (42,15)	**23,42**	32,87 (33,89)	30,47 (27,83)
Asparagus virus 2	**40,28**	42,93 (50,55)	**33,78**	38,06 (41,37)	**23,78**	32,65 (33,89)	30,13 (28,57)
Citrus variegation virus	**40,67**	43,36 (50,55)	**34,36**	39,46 (41,70)	**21,48**	33,02 (31,31)	31,30 (29,00)
Tomato necrotic streak virus	**39,93**	43,53 (51,77)	**33,08**	36,54 (39,03)	**22,25**	31,99 (34,23)	30,25 (28,33)
Average (% identity)	**40,01**	**43,20 (50,41)**	**33,86**	**37,89 (40,78)**	**22,41**	**32,49 (33,75)**	**30,91 (27,95)**
**Subgroup 3**							
Apple mosaic virus	**57,20**	53,72 (73,96)	**49,62**	49,02 (65,63)	**40,28**	48,71 (71,33)	48,71 (61,61)
Apple necrotic mosaic virus	**58,83**	53,60 (74,46)	**51,16**	49,62 (71,98)	**41,78**	46,69 (72,79)	50,28 (70,00)
Blueberry shock virus	**57,15**	53,57 (74,53)	**50,04**	51,37 (66,16)	**41,51**	50,60 (73,58)	50,20 (68,56)
Prunus necrotic ringspot virus	**58,94**	54,19 (76,05)	**50,95**	52,86 (67,73)	**48,14**	51,53 (73,61)	52,00 (68,72)
Lilac leaf chlorosis virus	**58,97**	53,86 (78,24)	**49,55**	49,03 (64,97)	**44,93**	52,99 (81,79)	49,65 (72,85)
Average (% identity)	**58,22**	**53,79 (75,45)**	**50,26**	**50,38 (67,29)**	**43,33**	**50,10 (74,62)**	**50,17 (68,35)**
**Subgroup 4**							
Fragaria chiloensis latent virus	**41,80**	44,58 (56,80)	**39,05**	38,54 (50,35)	**31,50**	35,19 (38,92)	36,15 (42,17)
Prune dwarf virus	**41,85**	45,90 (58,95)	**40,51**	42,15 (49,94)	**23,28**	38,34 (42,38)	32,32 (32,43)
Average (% identity)	**41,83**	**45,24 (57,88)**	**39,78**	**40,35 (50,15)**	**27,39**	**36,77 (40,65)**	**34,24 (37,30)**
**Unclassified**							
Humulus japonicus latent virus	**41,91**	44,95 (57,16)	**36,39**	36,52 (42,93)	**17,84**	35,32 (40,33)	28,63 (29,96)
American plum line pattern virus	**39,39**	42,66 (51,15)	**37,24**	36,08 (43,17)	**31,48**	30,82 (35,53)	34,34 (39,64)
Average (% identity)	**40,65**	**43,81 (54,16)**	**36,82**	**36,30 (43,05)**	**24,66**	**33,07 (37,93)**	**31,49 (34,80)**
