# Supplementary material for: The Virome of Babaco (Vasconcellea × heilbornii) Expands to Include New Members of the Rhabdoviridae and Bromoviridae
Source: Viruses. 2023 Jun 16;15(6):1380. doi: 10.3390/v15061380 (PMC10304175; doi:10.3390/v15061380)
Supplement: Supplementary file 1 [file viruses-15-01380-s001.zip › Supplementary Table S4.pdf]

Supplementary Table S4. List of ilarviruses used in phylogenetic analyses.

Name	Acronym	Replicase P1a	RdRp P2a	MP	CP
Ageratum latent virus	AgLV	YP_008470969.1	YP_008470970.1	YP_008470972.1	YP_008470973.1
American plum line pattern virus	APLPV	NP_602312.1	NP_602313.1	NP_602314.1	NP_602315.1
Apple mosaic virus	ApMV	NP_604469.1	NP_604470.1	NP_604484.1	NP_604485.1
Apple necrotic mosaic virus	ApNMV	YP_009551612.1	YP_009551615.1	YP_009551613.1	YP_009551614.1
Asparagus virus 2	AV2	YP_002455928.1	YP_002455929.1	YP_002455926.1	YP_002455927.1
Babaco ilarvirus 1	BabIV-1	OQ256238	OQ256239	OQ256240	OQ256240
Blackberry chlorotic ringspot virus	BCRV	YP_002308569.1	YP_002308570.1	YP_002308572.1	YP_002308573.1
Blueberry shock virus	BlShV	YP_008519304.1	YP_008519305.1	YP_008519306.1	YP_008519307.1
Cape gooseberry ilarvirus 1	CGIV-1	YP_009551515.1	YP_009551513.1	YP_009551516.1	YP_009551517.1
Citrus leaf rugose virus	CiLRV	NP_613282.1	NP_613281.1	NP_613279.1	NP_613280.1
Cucumber mosaic virus	CMV	NP_049323.1	NP_049324.1	NP_040776.1	NP_040777.1
Citrus variegation virus	CVV	YP_001285482.1	YP_001285483.1	YP_001285480.1	YP_001285481.1
Elm mottle virus	EMoV	NP_619576.1	NP_619575.1	NP_619577.1	NP_619578.1
Fragaria chiloensis latent virus	FClLV	YP_164801.1	YP_164802.1	YP_164804.1	YP_164805.1
Grapevine associated ilarvirus	GAIV	QIJ25693.1	QIJ25694.1	QIJ25696.1	QIJ25697.1
Humulus japonicus latent virus	HJLV	YP_054422.1	YP_054423.1	YP_054424.1	YP_054425.1
Lilac leaf chlorosis virus	LLCV	YP_009104367.1	YP_009104368.1	YP_009104372.1	YP_009104373.1
Prune dwarf virus	PDV	YP_611154.1	YP_611151.1	YP_611152.1	YP_611153.1
Parietaria mottle virus	PMoV	YP_006446.1	YP_006447.1	YP_006463.1	YP_006464.1
Prunus necrotic ringspot virus	PNRSV	NP_733823.1	NP_733824.1	NP_733825.1	NP_733826.1
Privet ringsport virus	PrRSV	YP_009165996.1	YP_009165997.1	YP_009165999.1	YP_009166000.1
Prunus virus I	PrV-1	QSG73629.1	QSG73630.1	QSG73632.1	QSG73633.1
Peanut virus C	PVC	AWC08303.1	AWC08304.1	AWC08306.1	AWC08307.1
Potato yellowing virus	PYV	QGL51777.1	QGL51778.1	QGL51779.1	QGL51780.1
Rosa ilarvirus-1	RIV-1	QPB74030.1	QPB74031.1	QPB74033.1	QPB74034.1
Solanum nigrum ilarvirus 1	SNIV-1	QGN75791.1	QGN75794.1	QGN75799.1	QGN75800.1
Strawberry necrotic shock virus	SNSV	YP_941474.2	YP_941472.1	YP_941470.1	YP_941471.1
Spinach latent virus	SpLV	NP_620677.1	NP_620678.1	NP_620680.1	NP_620681.1
Tulare apple mosaic virus	TAMV	NP_620753.1	NP_620754.1	NP_620756.1	NP_620757.1
Tomato aspermy virus	TAV	NP_620760.1	NP_620761.1	NP_620758.1	NP_620759.1
Tomato necrotic spot virus	TNSV	AYN45099.1	AYN45100.1	AYN45102.1	AYN45103.1
Tomato necrotic streak virus	ToNSV	YP_009508872.1	YP_009508870.1	YP_009508873.1	YP_009508874.1
Tea plant line pattern virus	TPLPV	YP_009551567.1	YP_009551568.1	YP_009551569.1	YP_009551570.1
Tobacco streak virus	TSV	NP_620772.1	NP_620768.1	NP_620773.1	NP_620774.1
Viola white distortion associated virus	VWDAV	ANS71064.1	ANS71063.1	ANS71062.1	ANS71061.1
Water chestnut virus A	WCVA	UCY33659.1	UCY33660.1	UCY33661.1	UCY33662.1
