# Supplementary material for: The Virome of Babaco (Vasconcellea × heilbornii) Expands to Include New Members of the Rhabdoviridae and Bromoviridae
Source: Viruses. 2023 Jun 16;15(6):1380. doi: 10.3390/v15061380 (PMC10304175; doi:10.3390/v15061380)
Supplement: Supplementary file 1 [file viruses-15-01380-s001.zip › Supplementary Table S5.pdf]

Supplementary Table S5. List of rhabdoviruses used in phylogenetic analyses.

Virus name	Abbreviation	Accession
Alfalfa associated nucleorhabdovirus	AaNV	UBX89820.1
Artemisia capillaris nucleorhabovirus 1	AcNRV1	UKL15221.1
Alfalfa dwarf virus	ADV	YP009177021.1
Alopecurus myosuroides varicosavirus 1	AMVV1	LN713933
Apple rootstock virus A	ApRVA	QBZ28538.1
Agave tequilana virus 1	ATV1	DAF42283.1
Babaco rhabdovirus 1	BabRV-1	OQ256237
Black currant nucleorhabdovirus 1	BCaRV	AUW36419.1
Barley yellow striate mosaic virus	BYSMV	KM213865
Colocasia bobone disease-associated virus	CBDaV	KT381973
Cereal chlorotic mottle virus isolate M	CCMoV	MW731536
Cardamom vein clearing nucleorhabdovirus 1	CdVCV	QJZ27984.1
Citrus chlorotic spot virus	CiCSV	QBK95541.1
Citrus leprosis virus N	CiLV-N	AQN78378.1
Clerodendrum chlorotic spot virus	ClCSV	AWT62671.1
Cnidium virus 1	CnV1	UGY70985.1
Coffee ringspot virus	CoRSV	KF812526
Datura yellow vein nucleorhabdovirus	DYVV	AKH61406.1
Eggplant mottled dwarf alphanucleorhabdovirus	EMDV	QYA72338.1
Green sichuan pepper nucleorhabdovirus	GSPNuV	AZN18347.1
Joa yellow blotch-associated virus	JYBaV	QUI75406.1
Lettuce big-vein virus	LBVV	AB075039
Lettuce necrotic yellows virus	LNYV	YP425092.1
Maize fine streak virus	MFSV	AY618417
Maize iranian mosaic nucleorhabdovirus	MIMV	ABA60889.1
Morogoro maize-associated virus	MMaV	YP010087201.1
Maize mosaic nucleorhabdovirus	MMV	QCS90262.1
Maize yellow striate virus	MYSV	KY884303
Northern cereal mosaic virus	NCMV	AB030277
Orchid fleck dichorhavirus	OFV	QDM58727.1
Peach virus 1	PeV1	QIQ60850.1
Persimmon virus A	PeVA	AB735628
Physostegia chlorotic mottle virus	PhCMoV	QUJ09420.1
Papaya virus E	PpVE	MH282832
Potato yellow dwarf virus	PYDV	ARJ54297.1
Red clover varicosavirus	RCaVV	MF918568
Rice stripe mosaic virus	RSMV	KX525586
Rice yellow stunt virus	RYSV	AB011257
Strawberry crinkle cytorhabdovirus	SCV	MH129615
Sonchus yellow net nucleorhabdovirus	SYNV	NP042286.1
Sowthistle yellow vein virus	SYVV	QJQ80127.1
Taro vein chlorosis virus	TaVCV	YP224083.1
Tomato yellow mottle-associated virus	TYMaV	KY075646
Vesicular stomatitis Indiana virus	VSIV	NP_041716.1
Wheat yellow striate virus	WYSV	YP010086818.1
Yerba mate virus A	YmVA	QID92311.1
